# Food Waste Management Employing UV-Induced Black Soldier Flies: Metabolomic Analysis of Bioactive Components, Antioxidant Properties, and Antibacterial Potential

**DOI:** 10.3390/ijerph19116614

**Published:** 2022-05-28

**Authors:** Jiaxin Lu, Yuwen Guo, Atif Muhmood, Zheng Lv, Bei Zeng, Yizhan Qiu, Luxi Zhang, Pan Wang, Lianhai Ren

**Affiliations:** 1 School of Ecology and Environment, Beijing Technology and Business University, Beijing 100048, China; lujiaxin@btbu.edu.cn (J.L.); guoyw11@163.com (Y.G.); zbbzlha@163.com (B.Z.); 2130051001@st.btbu.edu.cn (Y.Q.); zlx201102@163.com (L.Z.); 2 State Environmental Protection Key Laboratory of Food Chain Pollution Control, Beijing Technology and Business University, Beijing 100048, China; 3 Key Laboratory of Cleaner Production and Integrated Resource Utilization of China National Light Industry, Beijing Technology and Business University, Beijing 100048, China; 4 Institute of Soil Chemistry & Environmental Sciences, Ayub Agricultural Research Institute, Faisalabad 38000, Pakistan; atif_1534@yahoo.com; 5 China National Development and Reform Commission Business Environment Development Promotion Center, Beijing 100101, China; lvzheng198497@163.com

**Keywords:** *Hermetia illucens* larvae, food waste, black soldier flies, UV-induced, metabolomics, bioactive compounds

## Abstract

Food waste, as a major part of municipal solid waste, has been increasingly generated worldwide. Efficient and feasible utilization of this waste material for biomanufacturing is crucial to improving economic and environmental sustainability. In the present study, black soldier flies (BSF) larvae were used as carriers to treat and upcycle food waste. Larvae of the BSF were incubated with UV light for 10, 20, and 30 min at a wavelength of 257.3 nm and an intensity of 8 W. The food waste utilization efficiency, antioxidant assays, antibacterial activity, and bioactive metabolites without and with UV treatment were determined and compared. Results showed that the BSF larvae feed utilization rate was around 75.6%, 77.7%, and 71.2% after UV treatment for 10, 20, and 30 min respectively, contrasting with the non-UV induced group (73.7%). In addition, it was perceived that the UV exposure enhanced antioxidant and antimicrobial properties of BSF extracts, and the maximum values were observed after 20 min UV induction time. Moreover, UV-induced BSF extracts showed an improved metabolic profile than the control group, with a change in the amino acids, peptides, organic acids, lipids, organic oxides, and other derivatives. This change in metabolomics profile boosted environmental signaling, degradation of starch, amino acids, sugars, and peptide metabolism. It was concluded that the bioconversion of food wastes using UV-induced BSF larvae can enhance the generation of a variety of functional proteins and bioactive compounds with potent antioxidant and antimicrobial activity. However, more studies are required to exploit the efficiency of UV treatment in improving BSF’s potential for upcycling of food wastes.

## 1. Introduction

Annually, one-third of global food production is wasted, equivalent to nearly 1.3 billion tons of unconsumed foodstuffs across the whole food supply chain. Furthermore, the worldwide economic effect of food waste is projected to be 750 billion (US) dollars each year [1]. Large amounts of food waste with high organic matter levels cause serious environmental pollution if discarded or disposed of improperly. Centralized food waste treatment, such as composting or anaerobic digestion technology, requires a significant investment in equipment, collection, storage, and transfer systems. Furthermore, the volatile composition, moisture content, and carbon to nitrogen ratio of food waste considerably influence the disposal efficiency. Therefore, there is a growing urgency to develop new and innovative technologies to treat food waste to facilitate its disposal, especially in developing countries. The Food and Agricultural Organization (FAO) has recommended edible insects being high protein animal feed as an alternative way of stabilizing feed prices and reducing environmental restrictions [2].

The black soldier fly (*Hermetia illucens*, BSF) is an insect found across tropical, subtropical, and mild temperate zones. Organic waste, such as decaying plant or animal parts, human or animal dung, food waste, abattoir waste, wine lees, sludge, and so on, can be biotransformed by BSF larvae [3]. It is regarded as a feasible and economically viable alternative because of its cost-effectiveness, ease of operation, low maintenance needs, and little imprint on the environment compared to other conventional technologies [4]. The BSF is considered one of the most promising species for large-scale production due to its high lipid content (48% of dry matter), high insect protein content (50% of dry matter), and the performance of manure as plant fertilizer [4]. Previously, BSF larvae were usually employed in traditional applications, such as biodiesel and animal feed. Cho et al. (2020) assessed the growth of BSF reared on food waste containing plastics and observed that although the weight of BSF larvae was improved than that of the control group, the growth rate of BSF adversely affected by the plastics in the food waste [5]. Similarly, it was found that the BSF survival rate was not influenced by the moisture content of food waste [4]. However, optimizing the food waste utilization by BSF still not well-considered, especially for changes in the bioactive compounds and metabolomic profiling of BSF feeding on food waste.

Technological advancements, such as improved induction and feeding conditions, can substantially enhance BSF larvae’s waste treatment efficiency. By activating the BSF larvae’s immune system, the larvae may become more efficient in treating food waste and produce more high-value bioactive compounds, such as active peptides and alkaloids, which have significant potential for use as antioxidants or in cosmetics. Among induction techniques, UV stimulation can activate the insect’s immune system, triggering hormonal and cellular defense responses and causing the generation of antimicrobial peptides in the insect [6]. Moreover, the interaction between ultraviolet stimulation and hormone signaling in BSF and the control of insect growth and development to manage food waste consumption is mainly unknown, and molecular research elucidating is still somewhat limited. Moreover, Oonincx et al. observed that UV-treatment stimulated vitamin D3 and D2 production in BSF and the type and contents depend upon the UV irradiance and exposure duration [7].

Metabolomics has emerged as a powerful analytical tool that has been widely applied to characterize the molecular response of many systems and organisms to external stimuli [8]. Melis et al. used metabolomics to investigate the effects of industrial freezing and drying processing regulation on yellow mealworm larvae’ metabolic pathways and nutritional properties [9]. Therefore, applying BSF’s metabolomics and revealing its metabolic pathways and molecular mechanisms is feasible and promising.

To address this knowledge gap, an untargeted LC–MS metabolomics approach was used herein to analyze UV-induced BSF larvae’s efficiency in the management and bioconversion of food wastes. The specific objectives of the study were (1) to improve the growth performance of the worms to maximize the consumption of food waste; (2) to identify and extract the bioactive compounds in the BSF and detect their antioxidant properties; and (3) to investigate the metabolic pathway of UV-induced BSF.

## 2. Results

### 2.1. Food Waste Reduction Potential of UV-Induced BSF

**Figure 1:**
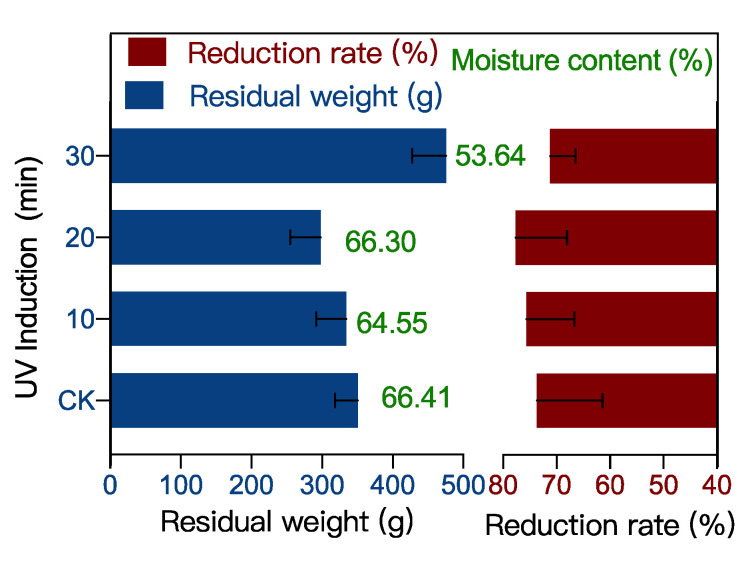
Amount of remaining food waste and the reduction of food and water reduction at UV-induced BSF versus the control (CK) after 5 days of feeding food waste (*p* < 0.05).

The remaining food waste in the control (CK) group was 351 g, with a water content of 66.4% and a reduction rate of 73.7% (Figure 1). The remaining undigested food waste by BSF after 10, 20, and 30 min of UV induction was 334 g, 298 g, and 476 g, respectively, with a water content of 64.5%, 66.3%, and 53.6% and a reduction rate of 75.6%, 77.7%, and 71.2%, respectively. Notably, UV-10 and UV-20 induced BSF digested the food waste more efficiently than the CK group, which may be due to the stimulation of the immune system of the BSF by UV treatment [10]. UV irradiation causes oxidative stress in insects [11]. BSF increases feeding to counteract the damage of oxidative stress and thus consumes more food waste [12]. However, when the UV induction mode was increased to 30 min, the rate of food waste digestion by the BSF decreased, which may be due to over-stimulation. Stimulation of the immune system can promote acetylcholine distribution in insects, thus promoting their vital activity [13]. While the over-stimulation can cause an excessive accumulation of acetylcholine that inhibits insect’s activity [14]. Therefore 10–20 min of UV stimulation can enhance the digestion of food waste by BSF, but 30 min of UV stimulation have an inhibitory effect on the digestion of food waste by BSF.

### 2.2. Antioxidant and Antimicrobial Activity of UV-Induced BSF Extract

**Figure 2:**
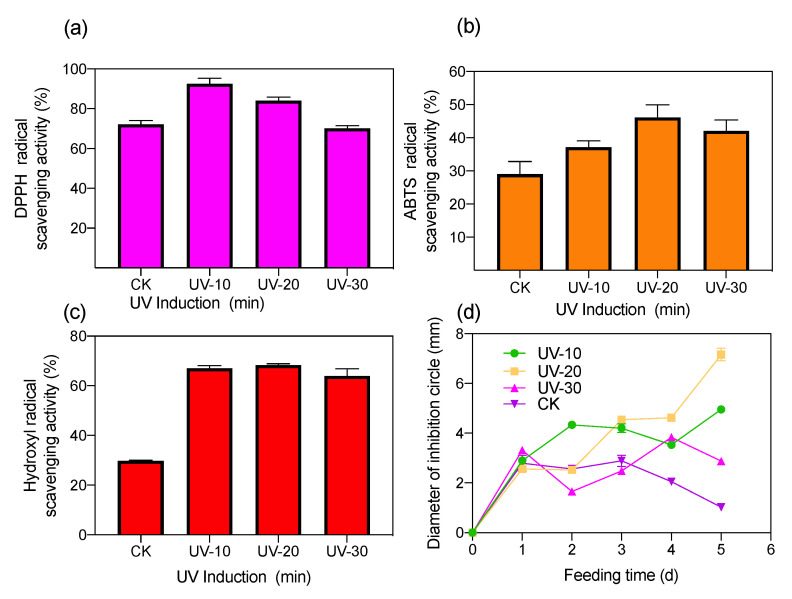
Comparison of antioxidant and antibacterial properties of UV-induced insect powder extracts from BSF feeding on food waste (**a**) DPPH, (**b**) ABTS, (**c**) Hydroxyl radicals, and (**d**) Antibacterial properties (*p* < 0.05).

The antioxidant activity of BSF under varying UV induction times can be explained by quantifying its ability to scavenge DPPH, ABTS, and hydroxyl radicals [15]. The DPPH, ABTS, and hydroxyl radical scavenging rates of uninduced BSF were 72.2%, 29.1%, and 29.8%, respectively (Figure 2). The maximum DPPH scavenging rate (92.6%) was observed after 10 min of UV treatment. It was further found that the DPPH scavenging rate decreased with an increase in UV treatment time. The maximum ABTS and hydroxyl radical scavenging activity of BSF (46.08% and 68.31%, respectively) were recorded after 20 min of UV induction (Figure 2b,c). This indicated that the antioxidants of the BSF extract were more capable of reversing ABTS cation formation and scavenging hydroxyl radicals after a 20-min UV induction. The increased scavenging capacity of antioxidants is mainly due to the formation of stable polysaccharide molecules and -OH active structural components that terminate free radical chain reactions. The improved radical scavenging activity of ABTS indicates that protein hydrolysates and peptides have enhanced radical scavenging activity. Furthermore, hydroxyl radicals are the most reactive radicals and can react with most biomolecules, including peptides, proteins, lipids, and DNA [16]. Therefore, after the UV treatment of BSF, the insects’ composition of proteins, lipids, and peptides changed. This is probably because more -OH structurally active components were produced in the insect body, which enhanced the radical scavenging activity against DPPH, ABTS, and hydroxyl radicals, thereby enhancing the antioxidant activity in BSF [17].

The antimicrobial activity of BSF increased with UV induction, and the highest inhibitory activity (with an inhibition circle diameter of 7.16 ± 0.26 mm) of the antimicrobial peptide after UV treatment for 20 min (Figure 2d). Several studies have demonstrated the antibacterial activity of BSF larvae against numerous pathogens, such as *S. aureus*, *S. epidermidis*, *B. subtilis*, and methicillin-resistant *S. aureus*, due to the presence of several haemolymphatic peptides as well as antimicrobial peptides in BSF [18]. UV induction enhances the BSF antimicrobial activity, mainly due to UV induction’s biological effect, which induces changes in DNA expression [19]. Furthermore, the formation of thymine dimers between BSF DNA duplexes and adjacent inter-thymidine dimers on the same strand by UV treatment prevents normal base pairing and incorrect replication [20]. This situation leads to a stop or change in the order of bases on the newly formed strand, thus causing mutations, which stimulate BSF to produce different antimicrobial substances [21].

### 2.3. Metabolic Profiling of UV-Induced BSF Extracts

#### 2.3.1. PCA and PLS-DA Analysis of the BSF with and without UV Treatment

LC-MS data were classified using chemometric methods to reduce dimensionality and improve interpretability to investigate UV induction’s effect on BSF metabolism. To visually present the overall clustering trends between UV-induced BSF and controls, PCA and PLS-DA were applied to the metabolite analysis of BSF samples. Possible differences between samples were minimized by unit variance (UV) and Pareto (Par) scales. PCA score plots (Figure S1) show QC samples clustered together (R^2^X = 0.739, Q^2^ = 0.264), indicating high stability and reproducibility of the instrument.

**Figure 3:**
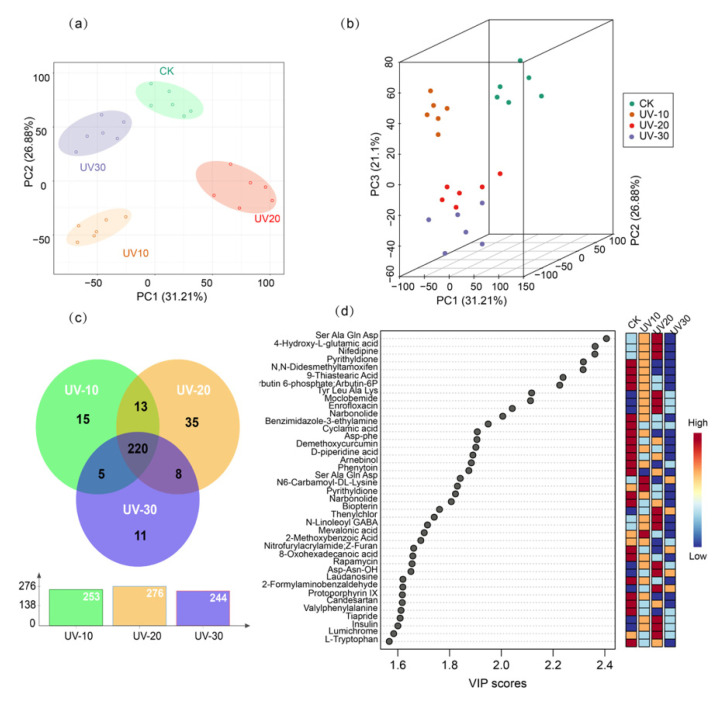
Multivariate analysis of UV-induced metabolites of BSF at different times: (**a**) 2D PCA scores (R^2^Y = 0.736, Q^2^ = 0.472), (**b**) PLS-DA 3D scores (R^2^Y = 0.935, Q^2^ = 0.73), (**c**) Venn diagram of differential metabolites between the treatment and control groups at different UV induction times (UV 10 min VS CK, UV 20 min VS CK, UV 30 min VS CK), and (**d**) specific differential metabolites between experimental and control groups and their VIP values.

Unsupervised PCA can project data from a high-dimensional space to a low-dimensional space without initial assumptions about its distribution [22]. As indicated in Figure 3a, the PCA scores of the samples fitted well (R^2^Y = 0.736) and showed good predictive values in the PCA score plot (Q^2^ = 0.472), with the first principal component explaining 26.9% of the variables and the second principal component explaining 31.2% of the variables. The unsupervised PCA plots show the relative clustering of samples from the same treatment, with a clear degree of separation between the UV-induced and control groups. This variation reflects the change in metabolites after digestion of food waste by BSF under varying UV induction times. After excluding outliers observed in the PCA model, a PLS-DA analysis was performed. PLS-DA is used for differentiating samples by reducing matrix dimensionality and maximizing the correlation between variables [23]. The difference in metabolite level between treatment PLS-DA was used to maximize the distinction between the UV-induced and control groups. The principal components generated by the PLS-DA analysis contained about 21.1% and 31.3% variables with R^2^Y and Q^2^ were 0.935 and 0.73, respectively (Figure 3b). The training set of LC–MS data showed a clear distinction between the UV-induced and control groups. As shown in Table 1, 330 significantly different metabolites were detected in the UV-induced BSF extracts (*p* < 0.05), containing 228 up-regulated and 102 down-regulated metabolites. Three categories of bioactive compounds, peptides, alkaloids, and fatty acids, were screened and summarized from the significant components and adjusted for Log_2_fold change ≥ 1.0-fold and Log_2_fold change ≤ −1.0-fold *p* ≤ 0.05. After UV induction, the abundance of small-molecule peptides such as dipeptide and tripeptide (-)-α-Kainic acid, Tyr-Leu, and Lysyl-Methionine were increased. In contrast, large molecule peptides such as Isoleucyl-Phenylalanine, Hydroxypropyl-Leucine, Asn Thr Gln Glu, and L-Valine gradually decreased. UV induction increases the number of dipeptides in BSF, probably due to a dominant role of innate immunity. A complex genetic cascade reaction is activated, culminating in the synthesis of a series of antimicrobial peptides and their release into the haemolymph after UV stimulation [24]. After UV induction, the increase in organic acids indicates that BSF can digest cellulose from food waste. Zhan et al. reported the BSF genome had experienced considerable development in functional modules that improved BSF’s adaptation to adverse environments, including immune system elements [25]. The immune system of BSF could be adapted to alleviate pathogens. Additionally, more produced peptidoglycan identification proteins within the genomes of BSF compared to other dipteran species have existed [26]. The peptidoglycan identification proteins mainly regulate signaling pathways during microbial infection [27]. The BSF genome also translates 50 antimicrobial peptides, making BSF the most excellent antimicrobial peptide creating a family in insects [28].

#### 2.3.2. OPLS-DA Analysis of BSF with Different UV Induction Times

OPLS-DA modelling of the data was carried out to investigate the effect of different UV induction times on the metabolism of BSF (Figure S2). The OPLS-DA analysis performed pairwise metabolite analysis, including UV 10 min vs. CK, UV 20 min vs. CK, and UV 30 min vs. CK. Differential metabolites of BSF were screened using the S-plot method. S-plot is a scatter plot that combines the covariance and correlation loadings distributions generated by a projection-based model, which visualizes the effects of the model’s variables [29]. Around 263, 276, and 244 significant metabolites were found in the UV-induced 10 min, 20 min, and 30 min groups, respectively, and 220 metabolites were covaried at different times of UV induction (Figure 3c). The results of the significant metabolite analysis were further compared by using hierarchical cluster analysis of the different UV induction time groups and the CK group. As shown in Figure S3, there was a clear color separation of the differential metabolites between the groups. The UV induction treatment showed a significant effect on the metabolism of BSF compared to the CK group.

Based on the experience of previous studies, multivariate variables affecting projection (VIP) values were considered the most useful for distinguishing treatment groups from controls, as a selection of metabolites is an important process for more accurate classification [30]. The top 40 significant metabolites with VIP > 1.0 were selected (Figure 3d). Metabolites that changed significantly in the BSF after UV induction include: amino acids, peptides and their derivatives (Ser Ala Gln Asp, Tyr Leu Ala Lys, L-Tryptophanamide, Asp-phe, Asp-Asn-OH, Ser Ala Gln Asp, Insulin); organic acids and their derivatives (4-Hydroxy-L-glutamic acid, Cyclamic acid, N-Linoleoyl GABA, N6-Carbamoyl-DL-Lysine, Valylphenylalanine; lipids and derivatives 9-Thiastearic Acid, Mevalonic acid, 8-Oxohexadecanoic acid, Narbonolide); organic oxides (Arbutin 6-phosphate; Arbutin-6P, Arnebinol, 2-Propylbenzimidazole); benzene and its substituents (Moclobemide, 2- Methoxybenzoic Acid, 2-Formylaminobenzaldehyde, Candesartan, Tiapride, N, N-Didesmethyltamoxifen); heterocyclic substances (Nifedipine, Pyrithyldione, D- piperidine acid, Nitrofurylacrylamide, Laudanosine, Protoporphyrin); and signal transducing hormones (N, N-Didesmethyltamoxifen, Biopterin, Lumichrome, Phenytoin, Mocimycin, Mocimycin). In general, by analyzing the metabolite content and composition of BSF at different UV induction times, it can be concluded that the metabolic changes in the BSF are closely related to UV induction. Overall, BSF larvae can acquire nutrients for their metabolic needs from the feed. BSF larvae utilize the carbohydrates monomer (glucose) as a tissue-building agent and energy [31]. Fly larvae are also surrounded by the carbohydrate chitin (Cohen, 2005). 

#### 2.3.3. Effect of UV Induction on the Enriched Metabolic Pathway of BSF

**Figure 4:**
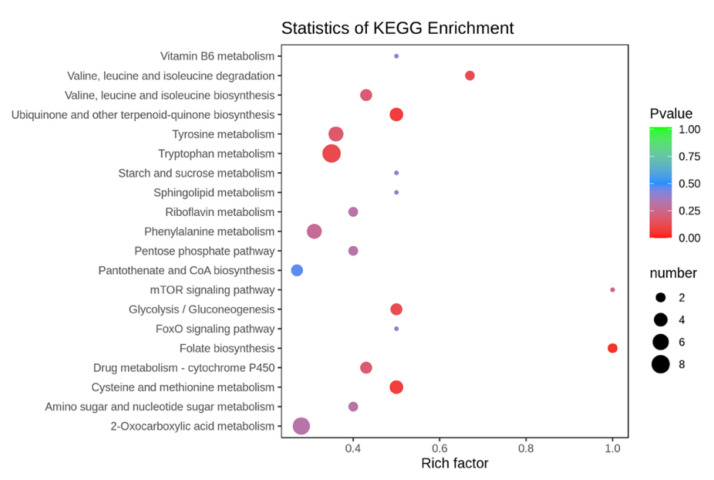
UV induction on the first 20 metabolic pathways of BSF, according to the Kyoto Encyclopedia of Genes and Genomes (KEGG) analysis. (*X*-axis enrichment factors, *Y*-axis metabolic pathway representation).

MetaboAnalyst 4.0 was used to enrich the metabolite data from the UV-induced and CK groups. According to the KEGG (Kyoto Encyclopedia of Genes and Genome) enrichment analysis, the 47 enrichment pathways were classified into five main categories (Figure 4), including environmental information processing pathways, insect organismal and metabolic pathways (carbohydrate metabolism, amino acid metabolism and lipid metabolism), genetic information processing pathways, and cofactor metabolism pathways. Based on effect values > 0.1 and −log(*p*) > 2 (*p* < 0.01), Figure 4 illustrates the top 20 metabolic pathways, mainly including the processing of environmental information (mTOR signaling pathway, FoxO signaling pathway), lipid metabolism (Sphingolipid metabolism,) cofactor metabolism (Vitamin B6 metabolism, Ubiquinone and other terpenoid-quinone biosynthesis, Riboflavin metabolism, Folate biosynthesis), amino acid metabolism (Valine, leucine and isoleucine degradation, Tryptophan metabolism, Tyrosine metabolism, Phenylalanine metabolism, cysteine and methionine metabolism), and digestive metabolism (Starch and metabolism of the digestive system (Starch and sucrose metabolism). This suggests that BSF may have first undergone environmental information transduction metabolism after UV induction to produce signaling molecules [32]. These molecules may have acted as messengers to stimulate the digestion of starch and sugars in food waste by the BSF digestive system [33]. This situation affects BSF metabolism and energy metabolism, including amino acid metabolism, lipid metabolism, and cofactor metabolism, further promoting the overall ability of BSF to produce bioactive compounds [34].

## 3. Discussion

### 3.1. Environmental Signaling Effects of UV-Induced BSF

**Figure 5:**
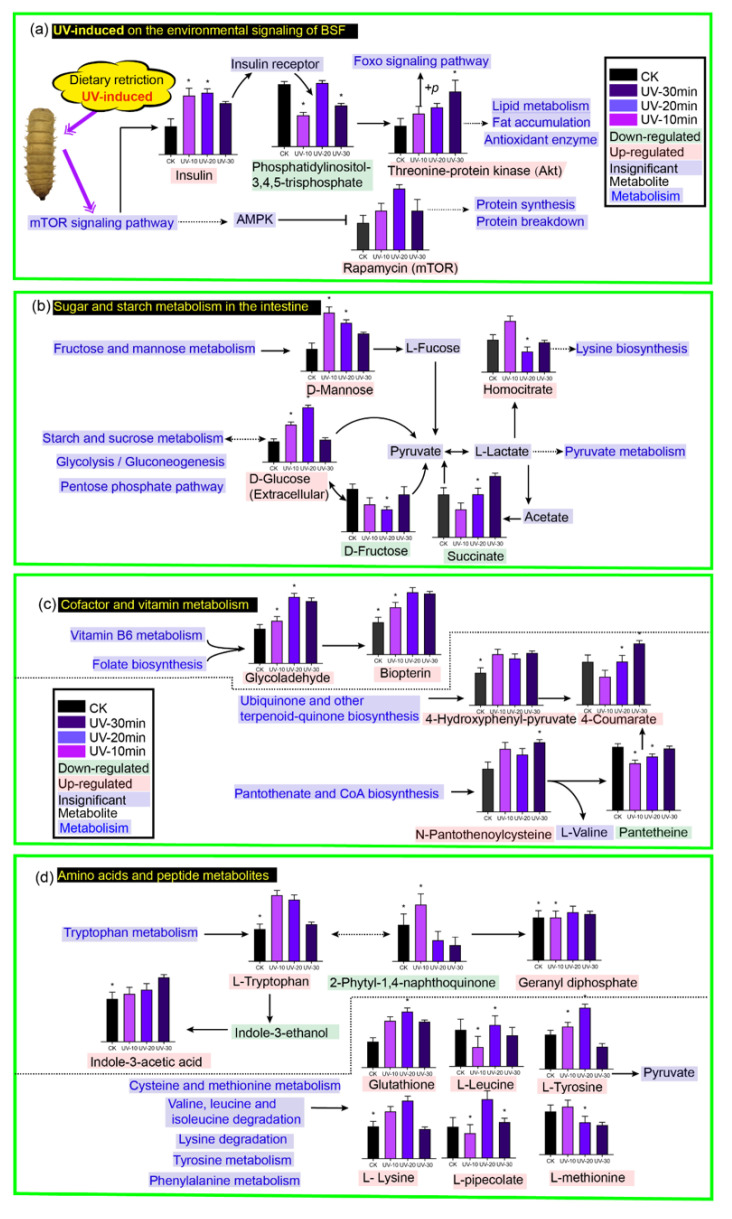
Effect of UV stimulation on metabolic pathways of BSF (**a**) environmental signaling, (**b**) enhanced degradation of starch and sugars from food waste, (**c**) cofactors for functional component production, and (**d**) amino acid and peptide metabolism (*p* < 0.05).

As shown in Figure 5, the UV-induced group showed a gradual increase in insulin levels, with its relative abundance rising from 895 in CK to 865, 1956, and 1625 in UV-10, UV-20, and UV-30, respectively. Rapamycin (mTOR) production was also enhanced, with its relative abundance rising from 865 in CK to 1253, 1953, and 1249 in UV-10, UV-20, and UV-30, respectively. The mTOR is regularly employed as a signaling, signaling stimulus, and immune regulator in different metabolic stress adaptations [35]. The mTOR regulates many processes required for cell growth and metabolism in the BSF, integrating cellular nutrition and stress status and inducing appropriate cellular responses. In addition, mTORC1 plays an important role in insect adipogenesis [36]. The mTOR signaling pathway can be activated by growth factors, amino acids, energy status, stress, and oxygen levels and produce signaling molecules to regulate various life processes such as predation and reproduction [37]. Thus, enhancing insulin induced by UV-induced BSF activates the mTOR signaling pathway. Subsequently, the vital activities of BSF (including lipid metabolism, proteolysis and synthesis, ribosomes, and amino and nucleotide sugars) are regulated, promoting the rate of catabolism of food waste and the conversion of bioactive compounds; however, excessive UV stimulation UV-30 leads to a decrease in insulin with corresponding UV damage.

UV induction resulted in a significant increase in Akt (threonine–protein kinase) in BSF, with its relative abundance increasing from 869 in CK to 1253, 1452, and 1952 under UV-10, UV-20, and UV-30. After phosphorylation, Akt can facilitate the production of the Forehead box O (Foxo) signaling pathway. FOXO can act as a terminal transcription factor of the insulin signaling pathway and regulate various physiological processes in many organisms, including insect longevity. Furthermore, the ATP-binding cassette (ABC) transporters’ metabolic pathway of BSF was enhanced by UV induction, providing energy for various anabolic processes. The UV-induced BSF extracts showed enhanced Pyrimidine and Purine metabolism, resulting in the production of Thymidine and Adenosine. Thymidine is a glycosaminoglycan molecule that stimulates fibroblast growth and promotes wound healing [38]. On the other hand, adenosine is an important signaling molecule involved in the stress response. It is one of the key signaling molecules that contributes to cytoprotection, immune response, regeneration, and balancing energy metabolism [39]. Ado signaling affects various physiological processes, including the regulation of synaptic plasticity, the proliferation of intestinal stem cells, differentiation of blood cells and metabolic adjustments during immune responses [40]. The terpenoid backbone biosynthesis pathway of BSF is enhanced by UV induction. Terpenoid backbone biosynthesis is associated with the biosynthesis of insect alarm pheromones that regulate insect life activities in response to oxidative stress, including insulin, glycolysis and isoprenoid pathways. Some researchers documented that in the existence of branched-chain amino acids, the BSF releases signals that create brain insulin-like hormones, which initiate metabolic processes, boosting larval growth.

### 3.2. Effect of UV Induction on the Digestion of Sugars and Starch in Food Waste by BSF

Food waste hydrolysis products are bioavailable for larval and/or microbial metabolism. UV induction played a facilitative role in the digestion of sugar and starch in food waste by BSF. The main metabolic pathways involved were amino sugar and nucleotide sugar metabolism, fructose and mannose metabolism, starch and sucrose metabolism, Glycolysis/Gluconeogenesis, and the Pentose phosphate pathway. Compared with the CK, the UV-induced BSF group was enriched in the significant metabolites D-mannose and D-glucose, whose relative abundance increased from 895 and 694 in CK to 2365 and 1256 in UV-10, 1971 and 1835 in UV-20, respectively. In insects, the digestion of dietary starch-reducing sugars is dependent only on amylase and maltase at the end of the insect midgut and up to the middle of the posterior midgut [41]. Fructose and mannose metabolism promotes the abundance of D-mannose and D-glucose, commonly found in insects, and is mainly used to support various energy-requiring functions. The UV-induced BSF enhanced the energy supply to the organism by facilitating the digestion of fructose and mannose. Glucose, a product of starch breakdown in food waste, is absorbed in the midgut of the BSF. D-glucose provides the reducing group for complex sugar or glycosidic linkages in the insect gut [42]. The elevated glucose demonstrated an increase in starch catabolism in the insect digestive tract, suggesting that UV induction facilitated the consumption of starchy material from food waste by BSF.

### 3.3. Effect of UV Induction on the Metabolism Cofactors and Bioactive Compounds in Food Waste by BSF

UV induction enhanced the ubiquinone and other terpenoid quinone biosynthesis pathways. UV induction promoted the production of 4-Hydroxyphenyl-pyruvate and 4-coumaric acid. 4-Coumarate is a CoA ligase that promotes the synthesis of flavonoid substances [43]. UV induction also facilitated the folate biosynthesis metabolic pathway of BSF and the production of biopterin, which acts as an endogenous enzyme cofactor in multi-animal species and some bacteria and fungi. Although insects cannot synthesize folic acid directly, bacteria that live in symbiosis with arthropods can induce folic acid (vitamin B9) biosynthesis, promoting energy storage and, thus, developing in insects [44]. CoA is a critical cofactor for various metabolic activities, including fatty acid production in insects. It also works as a cofactor for various enzymes involved in amino acid metabolism by synthesizing vitamin-like substances in the gut through diet. UV induction promotes the ability of BSF to absorb vitamins from the gut, which are often precursors to active coenzymes, thus promoting the insect’s vital activities. The extraction of B vitamins from microorganisms in the gut or tissues of insects has been studied [45].

### 3.4. Effect of UV Induction on Amino Acids and Peptides in BSF

Cysteine and methionine metabolism is enhanced in UV-induced BSF, promoting the relative abundance of glutathione. Glutathione is a tripeptide composed partially of glutamate, cysteine, and glycine. Glutathione can be present in reduced form (GSH) or as an oxidized dimer (GSSG), and the reduced equivalents of glutathione can play an important role in a variety of physiological processes in BSF [46]. Glutathione is a biological antioxidant that helps to protect cells from reactive oxygen species. L-Homocysteine is a protein building block required to manufacture keratin and collagen in insects. Tryptophan degradation occurred in BSF, with elevated indole-3-acetic acid in the UV-induced group, accompanied by Indole-3-ethanol degradation. A metabolic pathway from indole-3-ethanol to indole-3-acetic acid has been demonstrated in Drosophila [47]. Thus, UV induction enhances the conversion of indole-3-ethanol to indole-3-acetic acid (IAA) in BSF. IAA has been shown to play a significant role in promoting Wilkinson’s biochemical structures (lipids, proteins, glycogen, and lipid profiles) [48]. Tryptophan metabolism was enhanced by the UV induction of BSF, and geranyl diphosphate metabolites reached a maximum at 20 min of UV induction. Geranyl diphosphate promotes insect pheromone biosynthesis. 3-nitro-L-tyrosine is a biomarker of oxidative stress marker, identified as an indicator or marker of cellular damage inflammation. The highest levels of 3-nitro-L-tyrosine were found when UV was induced for 30 min, demonstrating that excessive UV exposure can be detrimental to the BSF. 

### 3.5. Application Potential of Bioactive Compounds in UV Induced BSF

The bioactive components detected in UV induced BSF relative to CK consisted mainly of peptides, alkaloids, and fatty acids (Table 1). Insect extracts rich in antioxidant active ingredients help prevent cell damage, limit free radical damage, are used as oxidants, or are used to develop cosmetic products, enhancing the economic benefits of the whole system of food waste- and BSF-high value products.

The relatively low toxicity of dipeptides is a significant advantage and is considered a promising feature in the field of antioxidants as well as cosmetics. Tyr-Leu (cyclo) has anxiolytic activity. Ac-Yvad-cho interferes with apoptosis initiation and helps slow down skin ageing. Glyphe is a dipeptide composed of phenylalanine and glycine with antioxidant activity. l-Valine can be used in supplemental dietary products for human and animal nutrition, pharmaceuticals, and as a moisturizing ingredient in skincare cosmetics. 2-Amino-3-phosphonopropionic acid, a glutamate receptor antagonist, can be used as an excitatory amino acid inhibitor to treat sensitive skins.

The alkaloid Piperolactam A can be used as a natural medicine in the production of cosmetics. Graveolinine can be used as an active compound to reduce inflammation, aid weight loss, and prevent heart and brain disease. Epigallocatechin increases lean body fat, and exercise activity promotes glucose metabolism and prevents oxidative stress. The steroidal ester Withaferin A has strong anti-diabetic, anti-inflammatory and anti-cancer effects. It may bind to the viral stinger (S-) protein of SARS-CoV-2 and could act as a potential therapeutic agent against COVID-19 infection [49]. Withaferin A can alter oxidative stress, promote apoptosis and autophagy, inhibit cell proliferation, and reduce angiogenesis and metastatic progression to attenuate various cancer markers. Hesperetin is a tyrosinase inhibitor with potential applications in medicine, cosmetics, and agriculture to prevent hyperpigmentation or browning.

Fatty acids are ω-hydroxy acid with ω-oxidized biological significance and have anti-inflammatory activity as well as biological activity to promote wound healing. (3R)-3- Hydroxydodecanoic fatty acid has significant antifungal properties. 20-Hydroxyeicosatetraenoic acid, also known as 20-HETE, has a wide range of effects on the vascular system, including the regulation of vascular tone, blood flow to specific organs, and the vascular pathway of remodeling [50]. Mandelic acid is widely used in cosmetics due to its properties, including exfoliation, disinfection, regulation of sebum production, antibacterial, acne skin, and skin-soothing. Isovaleric acid boosts immunity mainly by providing an important energy source for intestinal epithelial cells, inhibiting harmful bacteria by lowering intestinal pH, and indirectly regulating intestinal flora [51]. Organic acids in BSF can be used as skin care products, essential oils, medicines, colorants, and insecticides.

## 4. Materials and Methods

### 4.1. Materials

BSF eggs were obtained from Changzhou Weili Food Waste Treatment Plant (Changzhou, China). The food waste employed in this study was the separated residue from a three-phase oil extraction at Changzhou City’s Weili Food Waste Treatment Center. Food waste was collected continuously, combined, shredded, and kept at −20 °C. The food waste fed to BSF consisted mainly of vegetables, rice, and meat, and contained approximately 9.73% crude protein, 70.8% water, 5.80% crude ash, 1.98% crude fiber, 3.04% crude fat, and 9.73% starch. The food waste with a pH value of 7.16 used in the present study was measured with a pH meter (Thermo-868, Thermo Fisher Scientific, Waltham, MA, USA). The crude protein content of all food waste samples was determined using the Hach Digesdahl Method^®^ (Hach, Loveland, CO, USA) and reported as a percentage of dry matter. The incubation material was composed of wheat bran, peanut bran, and water in a mass ratio of 1:1:13 and had a moisture content of 67%.

### 4.2. Breeding Process

Freshly hatched BSF eggs were separated into 1–2 cm pieces and put in an extended brood. As bedding material, combined bran, pig’s blood, and water (2:1:1) were used in a 60 cm × 40 cm × 20 cm incubator. The split eggs (in the shape of lumps) were distributed in the incubator at a rate of 10 g/box. The eggs were kept warm by covering with bran at a moisture content of approximately 40% and the temperature was maintained at 32 °C with a relative humidity of 50%. After a three-day incubation period, the BSF had a hatching rate of over 80%. The feeding of BSF larvae continued for five days, and the larvae were transferred to the breeding workshop within 3 h of hatching on day five and the feeding of BSF with food waste was initiated.

### 4.3. UV Induction

**Figure 6:**
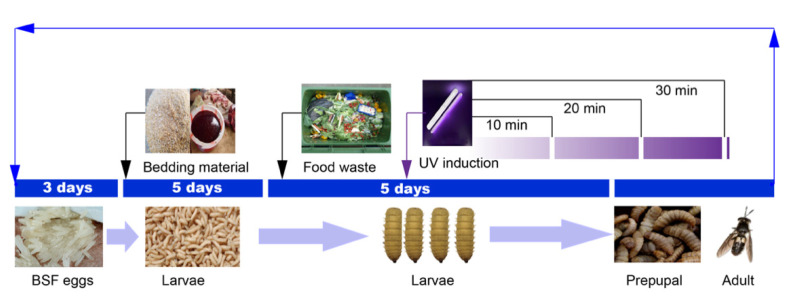
Schematic diagram of experimental design.

About 200 g of BSF larvae were placed in a square rearing box containing 1.25 kg of food waste. UV induction was performed on eight-day-old larvae by placing the plates under an 8 W UV lamp at a vertical distance of 35 cm for 10, 20, and 30 min, namely UV-10, UV-20, and UV-30, respectively (the UV wavelength was 257.3 nm, the power was 8 W, and the distance was 40 cm). A schematic diagram of the experimental design is shown in Figure 6. Three replicates were set for each treatment. Insect samples and food waste samples were taken daily for five days. The experiment was terminated when more than 90% of the larvae became prepupae, as the larvae stopped feeding when they became prepupae [4]. The proportion of prepupae was calculated daily by analyzing the biomass of 100 insect samples and calculating the number of dark brown samples as the morphological color of the BSF larvae changed from white to dark brown [52]. After feeding BSF for five days with food waste, prepupae emerged, followed by the harvesting of worms. The BSF were fasted for approximately 48 h to eliminate residual food from their gastrointestinal systems. Freeze-drying was carried out to eliminate moisture from the insects. For further investigation, approximately 100 g of BSF was freeze-dried, crushed, and stored at −18 °C.

### 4.4. Antioxidant Assays

To quantify antioxidant activity, 2,2′-diphenyl-1-picrylhydrazyl (DPPH) radical scavenging activity was determined by DPPH assay as described by Binsan with a slight modification [53]. An amount of 1 mL of BSF sample with an initial protein content of 0.2 mg mL^−1^ was mixed with 1 mL of DPPH-95% methanol solution, and the reaction was carried out for 30 min, then absorbance was measured at 515 nm. The cleaning effect was calculated using Equation (1).

(1) $$\mathrm{Scavenging}\;\mathrm{activity}\;\left(\%\right)=\frac{A_{\mathrm{control}}-A_{\mathrm{sample}}}{A_{\mathrm{control}}}\times 100$$

A_sample_ is the absorbance of the solution with the sample; A_control_ is the absorbance of the solution without the sample.

The capacity of BSF to scavenge 2,2′-Azino-Bis (3-Ethylbenzothiazoline-6-Sulfonic Acid) (ABTS) radicals was determined following the already reported procedure [54]. Briefly, equal volumes of ABTS solution and K_2_S_2_O_8_ were reacted in the dark for 12 h at 25 °C and then diluted with phosphate buffer (PBS) until their absorbance at 734 nm reached 0.7 ± 0.02. Then, 0.2 mL of sample (1 mg mL^−1^) was added to 0.8 mL of the working solution held at 25 °C for 5 min. The absorbance was measured at 734 nm to calculate the total antioxidant capacity. The capacity of BSF extract to scavenge hydroxyl radicals was determined using the following equation

(2) $$\mathrm{Scavenging}\;\mathrm{activity}\;\left(\%\right)=\frac{A_{\mathrm{control}}-A_{\mathrm{sample}}}{A_{\mathrm{control}}}\times 100$$

A_sample_ is the absorbance of the ABTs solution with the sample; A_control_ is the absorbance of the ABTs solution with 95% ethanol.

The sample, FeSO_4_, and salicylic acid-ethanol solution were mixed at a ratio of 1:1:1. Then 1.0 mL of H_2_O_2_ was added and incubated in a water bath at 37 °C for 30 min. Absorbance was measured at 510 nm using a spectrophotometer. Ascorbic acid was used as a positive control. The hydroxyl radical scavenging activity was calculated using the following formula.

(3) $$\mathrm{Scavenging}\;\mathrm{activity}\;\left(\%\right)=\frac{A_{1}-A_{2}}{A_{1}}\times 100$$

A_1_ is the absorbance of the sample; A_2_ is the absorbance of the solution without the sample.

### 4.5. Antibacterial Experiment

The antibacterial activity of the BSF sample against *Escherichia coli* was evaluated by the inhibition zone and inhibition ratio assays. *E. coli* were grown by shaking (200 rpm) at 37 °C in Luria–Bertani (LB) broth. A dilute solution of *E. coli* with OD600 = 1 was adjusted. Then, 100 µL of the solution was added to 5 mL of sterile water and shaken well. Around 500 µL of the former solution was aspirated, spread evenly on LB agar plates, and left for 10 min.

The BSF fractions were freeze-dried and dissolved in ultrapure water to a concentration of 50 mg mL^−1^. The amount of 10 µL of 50 mg mL^−1^ of different UV-induced BSF sample solutions and ultrapure water was added to a 6 mm sterile paper sheet and left to stand until the paper sheet had completely absorbed the solution. The culture medium was inverted and incubated in an incubator at 37 °C for 24 h. The diameter of the inhibition circle was then measured. Each treatment was replicated three times.

### 4.6. Untargeted Metabolomic Analysis

#### 4.6.1. Extraction of BSF

The BSF sample was resuspended in prechilled 80% methanol and 0.1% formic acid mixed with vortex shaken. After 5 min in an ice bath, the samples were centrifuged at 15,000 rpm at 4 °C for 10 min. The supernatant was diluted with LC–MS grade water to a final concentration of 60% methanol and then transferred to a new Eppendorf tube fitted with a 0.22 µm filter (Millipore, Bedford, MA, USA). The samples were then centrifuged for 10 min at 15,000 rpm. Finally, the filtrate was fed into the liquid chromatography–mass spectrometry apparatus for examination. The blank samples served as a blank matrix for the experimental samples, and the pretreatment process for the blank samples was similar to that for the experimental samples.

#### 4.6.2. Liquid Chromatography–Mass Spectrometry (LC–MS) Analysis and Data Processing

Extracts from each BSF sample were analyzed using a Nexera X2 LC-30A UFLC system utilizing a 100 mm 2.1 mm ACQUITY UPLC HSS T3 1.8 m HPLC column (Shimadzu). Metabolites were eluted from the column at a flow rate of 0.40 mL min^−1^ using a mobile gradient phase consisting of A (0.1% formic acid in water) and B (0.1% formic acid in acetonitrile). Initial conditions (5% B + 95% A), (90% B + 10% A) were maintained for 11 min, (90% B + 10% A) for 12 min, (5% B + 95% A) for 12.1 min, and (5% B + 95% A) for 14 min. For the collection of positive and negative data, a QTRAP 6500 + (AB SCIEX) and an electrospray ionization (ESI) source was used. The spray voltage was tuned to 4500 V and 3500 V for positive and negative ion modes. The temperature of the ion source was fixed at 650 °C. The ion source gases were tuned at a ratio of 1:50 and 2:50, respectively.

The relative abundance of metabolites was determined using a targeted metabolomics technique using an ultra-high performance liquid chromatography-tandem mass spectrometry system (UHPLC-MS/MS). MultiQuant 3.0.2 (ABSciex, Darmstadt, Germany) was used to examine the chromatograms and integrate the peak areas (Applied Biosystems SCIEX). To account for equipment or sample handling variances, the peak area of each identified metabolite was normalized to the total peak areas of all detected metabolites in the same sample. For subsequent analysis, the standardized area was employed as a variable. Quality control (QC) samples were aliquots of all test samples introduced at ten-sample intervals to evaluate the instrument’s stability throughout the operation. Earlier experiments included a systematic methodological validation. The prior validation findings showed that the method was sufficiently accurate and reliable. As a result, no internal standards were employed in this study’s tests.

#### 4.6.3. Identification of Metabolites

Compound Discoverer 3.0 (CD3.0, Thermo Fisher Scientific, Waltham, MA, USA) was used to handle the raw data files obtained from UHPLC-MS/MS for peak alignment, peak selection, and quantification of each metabolite. The following parameters were set: 0.1-min variance in retention time; 5 ppm mass deviation; 30% signal intensity deviation; 3 signal-to-noise ratios; and minimum signal intensity of 100,000. After normalizing the data, molecular formulas were predicted using extra ions, molecular ion peaks, and fragment ions. Finally, peaks were compared to the mzCloud (https://www.mzcloud.org/, accessed on 8 July 2021) and ChemSpider (http://www.chemspider.com/, accessed on 15 July 2021) databases in order to acquire correct qualitative and quantitative findings.

#### 4.6.4. Data Analysis

Following the assessment of insect feed metabolites, metabolites were analyzed using the Kyoto Encyclopedia of Genes and Genomes (KEGG) database (http://www.genome.jp/kegg/, accessed on 16 July 2021), the Human Metabolome Database (HMDB) database (http://www.hmdb.ca/, accessed on 16 July 2021), and the Lipidmaps database (http://www.lipidmaps.org/, accessed on 16 July 2021). Principal component analysis (PCA), partial least squares discriminant analysis (PLS-DA), and fold change (FC) analyses were performed on metaX (a flexible and comprehensive software for processing metabolomics data). Univariate analysis of variance (-test) was applied to calculate statistical significance (-value). The Bonferroni method was used to reduce the false discovery rate in this study. Metabolites with variable significance in the projection were considered differential metabolites. Volcano maps were used to filter metabolites of interest-based on metabolites and metabolites.

The data used for clustering heat maps were normalized using scores of differential metabolite intensity regions and plotted by the heat map package in the R language (version 3.5.1, R Foundation for Statistical Computing, Vienna, Austria). Correlations between differential metabolites were analyzed by COR in the R language (Pearson’s method). Statistically significant values of correlations between differential metabolites were calculated by cor. mtest in the R language. Considered statistically significant, correlation plots were drawn by the corrplot package in the R language. The functions of these metabolites and metabolic pathways were studied using the KEGG database. The enrichment of metabolic pathways for differential metabolites was performed when the ratios met the following conditions. Metabolic pathway enrichment was considered statistically significant when metabolic pathway values were <0.05.

### 4.7. Statistical Analysis

All data were obtained from three parallel experiments and expressed as mean ± SD and statistically analyzed using Graphpad prism 8.3. Data were considered statistically significant at *p* < 0.05.

## 5. Conclusions

In this study, the potential of UV-induced BSF for food waste management was explored. The untargeted metabolomics profiling and the antioxidant and antimicrobial activity of BSF were also evaluated. UV induction of BSF larva improved their food-utilizing efficiency, antioxidant assays, antibacterial activity, and bioactive metabolites. A total of 1375 metabolites were detected in the UV-induced BSF extracts compared to the uninduced control. Moreover, around 263, 276, and 244 significant metabolites (*p* < 0.05) were found in the BSF after UV induction for 10, 20, and 30 min. Similarly, the ABTS and hydroxyl radical scavenging activity was increased by 46.08% and 68.31%, respectively, after the 20-min UV treatment of BSF when contrasted with the control group. Furthermore, various bioactive compounds were detected in UV-induced BSF with antioxidant and antibacterial activity. The finding of this study will help in understanding the regulatory pathway of food waste management with UV-induced BSF and provide a valuable source for functional metabolomic studies.

## Figures and Tables

**Table 1:** Bioactive compounds screened from the significant components from different UV-induced treatments of BSF.

Category	Compounds	Index	Formula	Precursor (g/mol)		Abundance			Log_2_FC
					CK	UV-10	UV-20	UV-30	
**Peptides**	(-)-α-Kainic acid	MW0103805	C_10_H_15_NO_4_	212.09	37,437.47	56,806.2	39,603.8	47,494.9	1.006
	Tyr-Leu	MEDL00365	C1_5_H_22_N_2_O_4_	292.13	719.85	4842.0	865.97	4709.35	1.210
	Isoleucyl-Phenylalanine	MW0107571	C_15_H_22_N_2_O_3_	277.15	47,560.84	47,878.7	15,486	31,611.15	1.625
	Hydroxyprolyl-Leucine	MW0107402	C_11_H_20_N_2_O_4_	243.13	10,048.15	18,385.8	11,287.9	35,568.13	2.314
	Lysyl-Methionine	MW0108102	C_11_H_23_N_3_O_3_S	276.12	898.69	264,387	59,785	217,602.57	4.491
	Asn Thr Gln Glu	MW0145900	C_18_H_30_N_6_O_10_	489.20	157,233.05	3447.24	1390.97	2182.42	1.245
	Ile Val Leu Glu	MW0151650	C_22_H_40_N_4_O_7_	471.29	5930.95	1703.68	10,064.1	4905.41	1.766
	Ac-Yvad-cho	MW0144352	C_23_H_32_N_4_O_8_	491.21	20,644.29	272,409.3	68,677.8	64,529.24	1.506
	Ser Ala Gln Asp	MW0156626	C_15_H_25_N_5_O_9_	418.16	14,331.34	22,733.4	11,507.4	20,810.78	1.036
	Ac-DEVD-CHO	MW0144237	C_20_H_30_N_4_O_11_	501.18	56,303.65	84,072.9	91,302.4	77,289.35	2.309
	DL-Leucine	MEDP0752	C_6_H_13_NO_2_	130.08	38,200.09	35,940.6	28,033.1	47,911.62	1.480
	Gly-Phe	MEDN1029	C_11_H_14_N_2_O_3_	221.09	43,628.87	643,738	3,744,324	2,342,296.48	1.301
	L-Valine	MEDL00009	C_5_H_1_1NO_2_	116.07	92,603.12	7481.62	15,518.6	4595.11	1.193
	2-Amino-3-phosphonopropionic acid	MW0104504	C_3_H_8_NO_5_P	189.98	4635.25	64,681.7	24,372.4	59,307.2	1.139
**Alkaloids**	Piperolactam A	MW0000400	C_16_H_11_NO_3_	264.06	1507.38	4319.89	1329.52	1737.47	1.908
	Graveolinine	MW0124270	C_17_H_13_NO_3_	278.08	17,550.98	209,423	46,621.5	136,055.46	1.610
	Epigallocatechin	MEDL02039	C_15_H_14_O_7_	305.05	5480.58	187,292	952,427	614,951.93	1.129
	Withaferin A	MW0103132	C_28_H_38_O_6_	469.25	63,941.05	36,776.2	14,410.1	98,156.15	2.826
	Methylarmepavine	MW0115762	C_20_H_25_NO_3_	326.16	15,201.71	198,100	27,575.8	251,073.24	1.227
	Demethoxycurcumin	MEDL01854	C_20_H_18_O_5_	378.13	11,249.69	53,841.3	28,938.3	77,343.14	2.773
	Hesperetin	MEDL02106	C_16_H_14_O_6_	301.07	288,642.25	27,143.7	5930.95	8098.36	1.436
	Vitamin K	MW0103120	C_31_H_46_O_2_	449.32	26,053.19	34,390	97,277.2	22,556.79	-1.222
**Fatty acids**	Caffeic Acid	MEDP0302	C_9_H_8_O_4_	179.03	65,537.89	24,450.57	58,851.4	31,987.33	1.907
	Ganosporeric acid A	MW0053470	C_30_H_38_O_8_	525.25	13,253.72	103,550	17,550.9	82,876.28	−1.126
	13,14-dihydro-15-keto-tetranor PGF1	MW0141363	C_16_H_28_O_5_	299.18	66,761.84	6987.71	1777.06	3524.61	1.283
	(3R)-3-Hydroxydodecanoic acid	MW0103903	C_12_H_24_O_3_	215.16	176,791.1	167,876	37,938.4	60,404.19	1.987
	20-HETE	MEDP1153	C_20_H_32_O_3_	319.21	12,600.82	26,700.4	18,676.8	58,888.53	1.346
	Mandelic Acid	MEDN0334	C_8_H_8_O_3_	134.03	2371.76	3676.24	1132.86	4007.93	−3.041
	Isovaleric acid	MW0054155	C_5_H_10_O_2_	102.06	8064.84	33,994	49,511.4	34,828.08	3.111

## Supplementary Materials

The following supporting information can be downloaded at: https://www.mdpi.com/article/10.3390/ijerph19116614/s1, Figure S1: Distribution of PCA score plots regarding QCs (●) and other samples (●) showing clusters of QC samples aggregated (R^2^X = 0.739, Q^2^ = 0.264); Figure S2: OPLS-DA analysis of metabolic samples with extended UV induction times. (a–c) represent the 10 min, 20 min and 30 min groups, respectively, compared to the un-induced group. (d–f) are S-Plot plots obtained by comparing Con-1d, Con-3d, and Con-5d, respectively. The horizontal coordinates of the S-plot indicate the loadings of each substance on the first principal component, and the vertical coordinates indicate the correlation coefficient of each substance with the first principal component. Figure S3: Heat map of all metabolites identified by UV-induced BSF. Red color represents significant up-regulation of metabolite expression levels, while the blue color represents significant down-regulation compared to metabolite expression levels.
