# Supplementary material for: Food Waste Management Employing UV-Induced Black Soldier Flies: Metabolomic Analysis of Bioactive Components, Antioxidant Properties, and Antibacterial Potential
Source: Int J Environ Res Public Health. 2022 May 28;19(11):6614. doi: 10.3390/ijerph19116614 (PMC9179956; doi:10.3390/ijerph19116614)
Supplement: Supplementary file 1 [file ijerph-19-06614-s001.zip › ijerph-1719036-supplementary.pdf]

Food Waste Management Employing UV-Induced Black Soldier Flies: Metabolomic Analysis of Bioactive Components, Antioxidant Properties, and Antibacterial Potential

Jiaxin Lu [1,2,3], Yuwen Guo [1], Atif Muhmood [4], Zheng Lv [5], Bei Zeng [1], Yizhan Qiu [1], Luxi Zhang [1], Pan Wang [1,2,3,*] and Lianhai Ren [1,2,3,*]

1 School of Ecology and Environment, Beijing Technology and Business University, Beijing 100048, China; lujiaxin@btbu.edu.cn (J.L.); guoyw11@163.com (Y.G.); zbbzlha@163.com (B.Z.); 2130051001@st.btbu.edu.cn (Y.Q.); zlx201102@163.com (L.Z.)
2 State Environmental Protection Key Laboratory of Food Chain Pollution Control, Beijing Technology and Business University, Beijing 100048, China
3 Key Laboratory of Cleaner Production and Integrated Resource Utilization of China National Light Industry, Beijing Technology and Business University, Beijing 100048, China
4 Institute of Soil Chemistry & Environmental Sciences, Ayub Agricultural Research Institute, Faisalabad 38000, Pakistan; atif_1534@yahoo.com
5 China National Development and Reform Commission Business Environment Development Promotion Center, Beijing 100101, China; lvzheng198497@163.com
* Correspondence: wangpan@th.btbu.edu.cn (P.W.); renlh@th.btbu.edu.cn (L.R.)

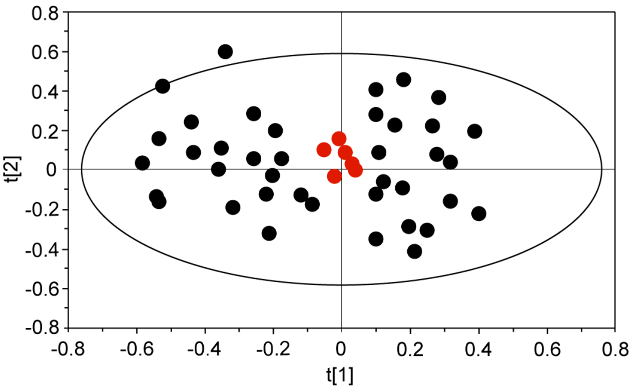

Figure S1. Distribution of PCA score plots regarding QCs (●) and other samples (●) showing clusters of QC samples aggregated ($R^2X = 0.739$, $Q^2 = 0.264$).

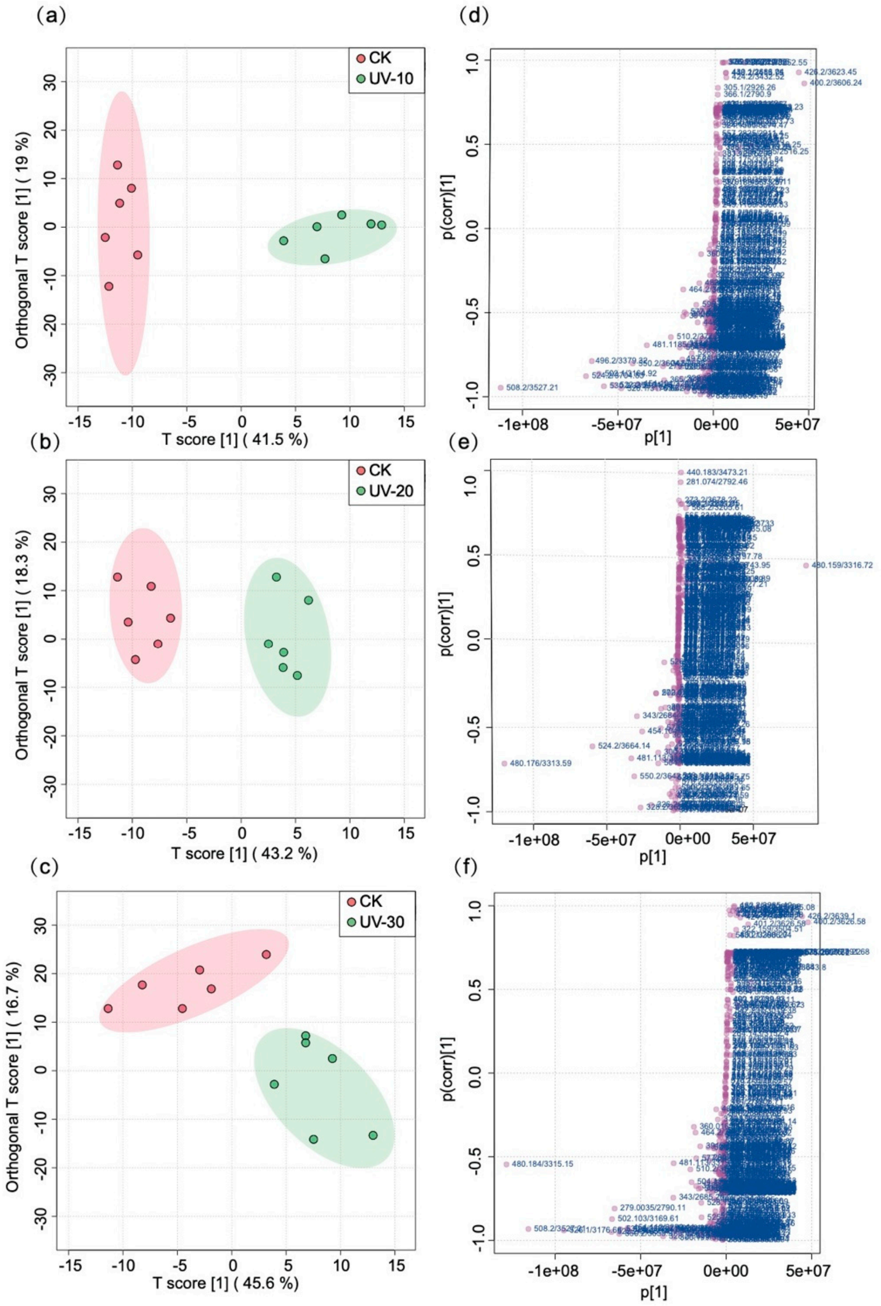

Figure S2. OPLS-DA analysis of metabolic samples with extended UV induction times. (a–c) represent the 10min, 20min, and 30min groups, respectively, compared to the un-induced group. (e–f) are S-Plot plots obtained by comparing Con-1d, Con-3d, and Con-5d, respectively. The horizontal coordinates of the S-plot indicate the loadings of each substance on the first principal component, and the vertical coordinates indicate the correlation coefficient of each substance with the first principal component.

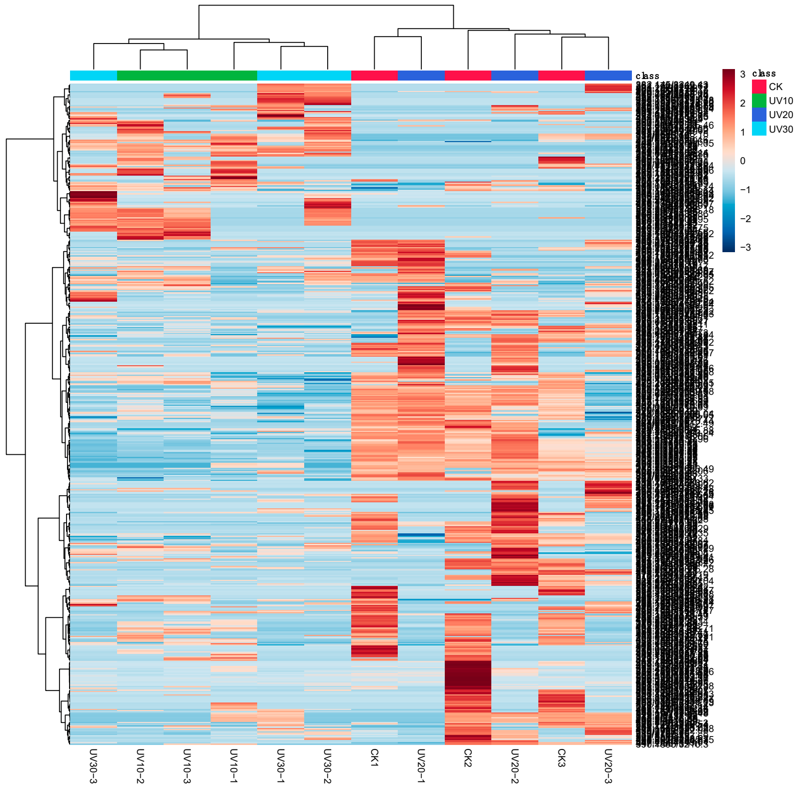

Figure S3. Heat map of all metabolites identified by UV-induced BSF. Red color represents significant up-regulation of metabolite expression levels; while the blue color represents significant down-regulation compared to metabolite expression level.
